# La luxation antérieure de l’épaule associée à une fracture de la diaphyse humérale homolatérale: à propos de deux cas

**DOI:** 10.11604/pamj.2017.28.293.12305

**Published:** 2017-12-05

**Authors:** Said Zizah, Kamal Lahrach, Amine Marzouki, Fawzi Boutayeb

**Affiliations:** 1 Service d’Orthopédie et Traumatologie A, CHU Hassan II, Fès, Maroc

**Keywords:** Fracture de l´humérus, luxation, épaule, homolatérale, Humeral fracture, shoulder dislocation, ipsilateral

## Abstract

L'association d'une luxation de l'épaule à une fracture homolatérale de la diaphyse humérale est une entité lésionnelle rare. Nous présentons deux cas de luxation antérieure de l'épaule avec une fracture humérale homolatérale concomitante. La réduction de la luxation de l'épaule suivie de fixation de la fracture de la diaphyse humérale par plaque vissée a été faite dans les deux cas. A quatre mois, la fracture de la diaphyse humérale était bien consolidée. Les deux patients ont repris leur activité à un an. La réduction primaire de la luxation de l'épaule suivie d'ostéosynthèse de la diaphyse humérale donne de bons résultats.

## Abstract (en)

The association of shoulder dislocation with fracture of the ipsilateral humeral diaphysis is a rare injury. We report two cases of anterior shoulder dislocation associated with concomitant ipsilateral humeral fracture. Reduction of shoulder dislocation followed by screw-plate fixation of the fracture of the humeral diaphysis was performed in both cases. At 4-months follow-up, the fracture of the humeral diaphysis was well consolidated. At 1-year both patients had returned to normal levels of activity. Primary reduction of shoulder dislocation followed by osteosynthesis of the humeral diaphysis yields good results.

## Introduction

L'association d'une luxation de l'épaule à une fracture homolatérale de la diaphyse humérale est une entité lésionnelle rare. Diverses méthodes de traitement de cette lésion complexe ont été proposées. Nous présentons deux cas de luxation antérieure de l'épaule avec une fracture humérale homolatérale concomitante et nous discutons le mécanisme lésionnel, les difficultés et les modalités thérapeutiques.

## Patient et observation

**Figure 1:**
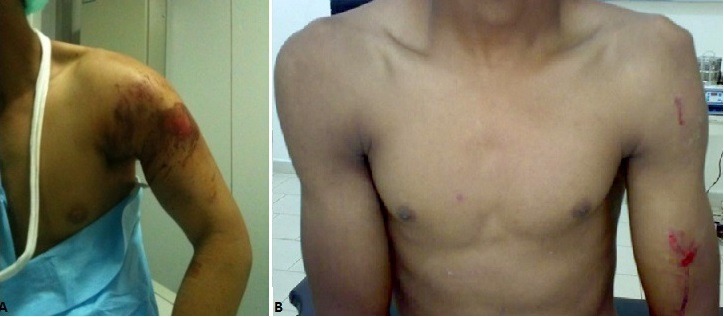
Aspect clinique avant traitement (A et B)

**Figure 2:**
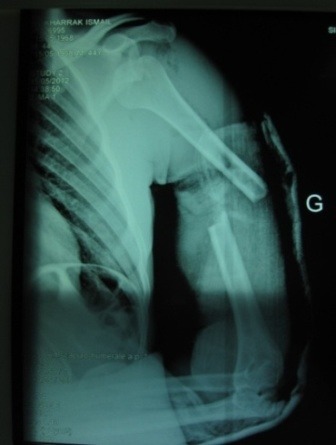
Radiographie de face de l’épaule montrant la luxation antérieure de l’épaule associée à une fracture de la diaphyse humérale homolatérale

**Figure 3:**
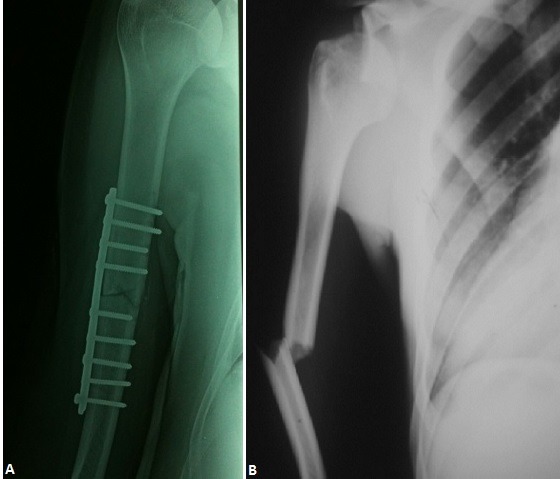
Contrôle radiologique après réduction de la luxation et fixation de la diaphyse humérale par plaque vissée (A et B)

**Première observation**: Un homme de 33 ans, maçon de profession, sans antécédents pathologiques notables, victime d'une chute d'une hauteur estimée à 2 mètres sur son côté droit. A l'admission, il était conscient et stable sur le plan hémodynamique. L'examen clinique a révélé une fracture ouverte stade I Cauchoix Duparc [1], des écorchures sur le bras sans déficit vasculonerveux. L'épaule droite était douloureux, enflés avec une perte du contour normal. Le bras était déformé en angulation (Figure 1A). Les radiographies ont montré une luxation antérieure de l'épaule avec une fracture transversale du tiers moyen de la diaphyse humérale homolatérale (Figure 2). Sous anesthésie générale, l'épaule a été réduite par une manipulation douce incluant séquentiellement: la rotation externe, une légère traction et de placement vers l'intérieur de la tête dans l'articulation scapulo-humérale (Figure 3A). Dans un second temps, on a procédé à une ostéosynthèse de la fracture de l'humérus par plaque vissée. Le membre a été immobilisé dans une écharpe coude au corps. Trois semaines après, le patient a commencé la rééducation pour récupérer les amplitudes articulaires avec des exercices de renforcement musculaire de l'épaule. La consolidation a été obtenue quatre mois après l'opération. A 3 ans de recul, l'épaule du patient était stable, indolore avec des amplitudes articulaires conservées. Aucun signe de lésions arthrosiques de l'épaule ou de tendinite calcifiante n'a été objectivé dans les dernières radiographies.

**Figure 4:**
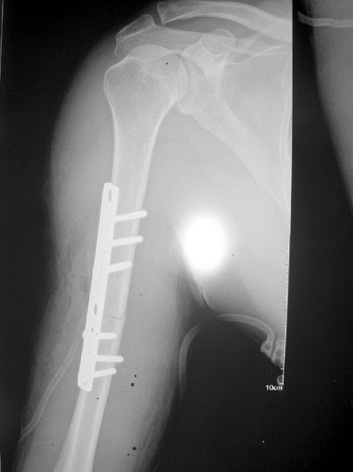
Radiographie d’une luxation antérieure de l’épaule associée à une fracture de la diaphyse humérale homolatérale

**Deuxième observation:** Monsieur HL, âgé de 34 ans, sans antécédent pathologique particulier, a été victime d'une chute dans une descente dangereuse, lors d'une compétition nationale de cyclocross, avec réception sur le membre supérieur gauche en hyperextension. L'examen clinique initial trouvait un patient conscient, stable sur le plan hémodynamique, présentant une attitude du traumatisé du membre supérieur avec saillie externe anormale de l'acromion (Figure 1B), sans déficit vasculonerveux, notamment pas d'atteinte du nerf radial. Le bilan radiologique objective une luxation antéro-interne de l'épaule associée à une fracture homolatérale de la diaphyse humérale (Figure 4). Comme pour le premier cas, la réduction de la luxation était réalisée en urgence sous anesthésie générale. Le contrôle radiologique retrouve une bonne congruence articulaire après réduction. Dans un second temps, on a réalisé une ostéosynthèse de la fracture diaphysaire de l'humérus par une plaque vissée (Figure 3B). L'épaule, jugé stable après réduction, était immobilisée dans une écharpe coude au corps pendant trois semaines, suivie d'une rééducation. A 45 jours, la fonction de l'épaule était comparable au côté controlatéral. L'épaule demeurait indolore, sans instabilité, avec des amplitudes symétriques au côté controlatéral. À trois mois, la fracture de la diaphyse humérale était bien consolidée. Le patient a repris son activité sportive à un an.

## Discussion

La luxation antérieure de l'épaule avec une fracture de l'humérus homolatérale est une lésion rare. Le premier cas dans la littérature moderne a été décrit en 1940 [2]. Depuis lors, 22 cas dans 17 journaux ont été rapportés par d'autres auteurs. Elle est la conséquence, en général, d'un traumatisme à haute énergie. Nos cas sont survenus lors d'une compétition sportive de cyclocross chez le premier malade et suite à une chute dans un accident de travail chez le deuxième malade. Beaucoup de travaux ont décrit le mécanisme des luxations antérieures de l'épaule [3, 4]. Le mécanisme décrit est similaire à celle du tableau de bord dans un accident d'automobile où une fracture de la diaphyse fémorale associée à une luxation de la hanche homolatérale [5]. Les mouvements d'abduction et de rotation externe dégagent la zone de faiblesse inférieure de la capsule et du ligament glénohuméral inférieur [6] qui ne sont plus renforcés par le muscle sous-scapulaire dans cette position (celui-ci passe en dessus). Ces mouvements sont associés à une force de torsion dans l'axe de l'humérus [7]. Selon Sankaran-Kutty [8], dans les cas de luxation antérieur de l'épaule et fracture de la diaphyse humérale, la force est transmise par l'axe de l'humérus à l'épaule. L'énergie est distribuée simultanément à l'humérus, qui se fracture, et à l'articulation de l'épaule. Diverses méthodes de traitement de cette lésion complexe ont été proposées. La réduction fermée et une attelle a été utilisée dans cinq cas avec de bons résultats obtenus dans 4 des 5 reprises [2, 7, 9]. La réduction fermée suivie d'une fixation externe a été également recommandé dans deux rapports [8, 10] avec des résultats satisfaisants. D'autre part, une ostéosynthèse par plaque a été appliquée dans sept cas produisant de bons résultats dans 5 d'entre eux, cependant, une paralysie du nerf radial dans un cas, et une lésion du plexus brachial dans un autre ont été enregistrées [9, 11, 12]. Dans nos cas, les deux malades ont bénéficiés d'une réduction de la luxation de l'épaule sous anesthésie générale suivie de la fixation de la fracture par plaque vissée avec bonne évolution. L'utilisation de broches a été décrite dans deux cas avec une évolution favorable et équitable [11, 12]. L'enclouage centro-médullaire a été utilisé dans certains cas [7] avec bons résultats fonctionnels. L'utilisation d'un fixateur externe [3], ou broches de Steinman [6] comme des outils de réduction, a été décrite. En outre l'ostéosynthèse par plaque vissée avant la réduction a été utilisée avec succès [13]. L'analyse de la littérature trouve deux conclusions majeures: la première est qu'il n'y a pas de consensus thérapeutique; la seconde est que dans un certain nombre de cas [ 1, 6, 8], la réduction fermée de l'épaule avant la fixation de fracture a échoué. Ce dernier est plus habituel dans les cas où le fragment proximal de la diaphyse de l'humérus est trop court pour permettre une manipulation adéquate [14]. Dans nos cas, la fracture de l'humérus a été fixée par une plaque vissée, suivie d'une immobilisation de l'épaule dans une écharpe pendant trois semaines. Le pronostic de ces lésions est relativement bon pour la plupart des auteurs [13, 15].

## Conclusion

La luxation antérieure de l'épaule associée à une fracture de la diaphyse humérale homolatérale est une lésion rare. Un traitement adéquat est indispensable pour éviter que des complications des deux lésions s'additionnent et compromettent la fonction du membre. Le pronostic fonctionnel de cette lésion dépend de celui de l'épaule d'où l'intérêt d'une bonne rééducation.

## Conflits d’intérêts

Les auteurs ne déclarent aucun conflits d'intérêts.
